# Balancing positive and negative luminescence for thermoradiative signatureless communications

**DOI:** 10.1038/s41377-025-02119-y

**Published:** 2026-03-05

**Authors:** Michael P. Nielsen, Stefan A. Maier, Michael S. Fuhrer, Nicholas J. Ekins-Daukes

**Affiliations:** 1 School of Photovoltaic and Renewable Engineering, UNSW Sydney, Kensington, 2052 NSW Australia; 2 School of Physics and Astronomy, Monash University, Clayton, 3800 VIC Australia; 3 Department of Physics, Imperial College London, London, SW7 2AZ UK

**Keywords:** Mid-infrared photonics, Fibre optics and optical communications

## Abstract

Ambient-temperature thermal infrared radiation is an underutilised resource for secure communications. Demonstrations of free-space data transfer using thermal radiation have been few, and have relied on intrinsically slow modulation of either the emissivity and/or physical temperature of a broadband blackbody emitter, severely limiting data transfer rates ( < 1 kHz). Here, we demonstrate a covert communications method in which photon emission is rapidly electrically modulated both above and below the level of a passive blackbody at the emitter temperature. The time-averaged emission can be designed to be identical to the thermal background, realizing communications with zero optical signature for detectors with bandwidth lower than the modulation frequency. We demonstrate this scheme using both electro- and negative luminescence in thermoradiative diodes, enabling data rates up to at least 100 kbps and modulation rates above 1 MHz. Future prospects for ultra-high-bandwidth (up to THz) emitters and detectors utilising meta-optics and 2D materials are discussed.

## Abstract (web summary)

We demonstrate covert transmission of data by rapidly modulating mid-infrared photodiodes, balancing forward and reverse bias luminescence. Only fast detectors resolve the signal; slow observers observe no evidence of communication.

## Graphical abstract

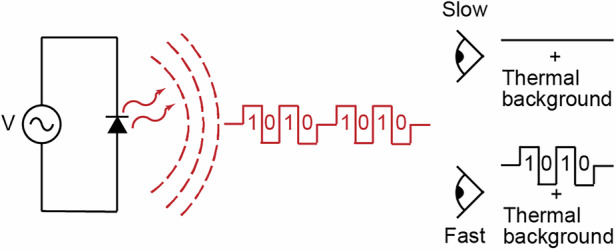

## Introduction

Covert communications, wherein the very act of communication is hidden and undetectable from an observer rather than merely encrypted, provides the most secure possible form of data transfer^1^. A classical example of this is steganography^2^, wherein information is concealed within another message or image. In optical communications, covert communications has been proposed via reducing the signature of emission so that to an observer it is indistinguishable from the background. One method is to hide the data transfer in an otherwise lossy and noisy channel, such as in the thermal noise from blackbody radiation^1^.

**Fig. 1:**
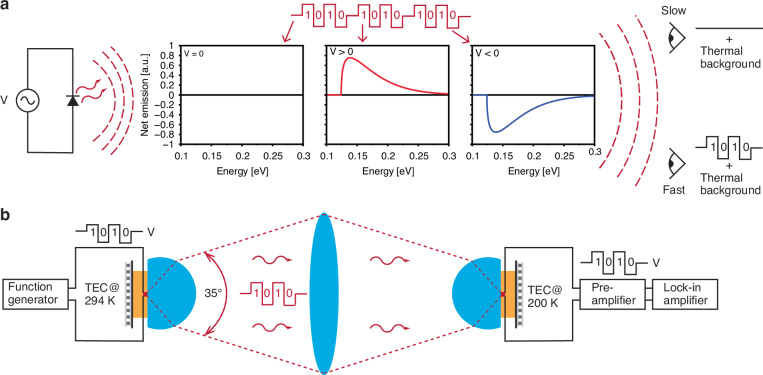
Conceptual depiction and experimental realisation of thermoradiative signatureless communication. **a** Conceptual depiction. A rapidly modulated voltage bias on a mid-infrared emitter (in this case a photodiode) imprints a signal that is either brighter than the thermal background (a binary ‘1’) or dimmer than the thermal background (a binary ‘0’), with only the observer who can resolve the signal fast enough able to distinguish it from the background. The plots show the net photon emission after thermal background subtraction from an ideal semiconductor with bandgap 0.124 eV at no bias (V = 0), forward bias (V > 0) and negative bias (V < 0). **b** Schematic representation of the experimental setup. The bias dependent emission from a mid-infrared photodiode at 294 K is imaged onto a nominally identically photodiode at 200 K and recorded

While typically optical communication occurs via additive (positive or “bright beam”) emission, and can be hidden in noisy optical channels^1^, another possibility for covert communication has been relatively unexplored: the use of subtractive (negative or “dark beam”) emission. Particularly, the combination of positive and negative emission can be utilised to make the time-averaged emission zero. In the case of thermal emission, this means the time-averaged emissions can be made to be identical to the thermal background, with zero signature for any detector with insufficient bandwidth. In any “bright” communications methodology, even if an observer does not yet have the method to resolve the communications, they will know the communication has occurred and can thus be prepared to acquire the means to resolve it going forward. With a covert method such as the one proposed here, the observer does not know that communication has occurred in the first place and thus may not deploy the methods necessary to resolve it. Such a covert technique is also still compatible with encryption techniques to further protect the communications. Recently, several groups have demonstrated free-space thermal data communications by controlling the emissivity and/or temperature of an object and used this to encode information on its thermal emission wherein the time-averaged signal blends into its environment^3–5^. This allows for free-space optical data transfer via the 3–5 µm and 8–14 µm atmospheric transmission windows. However, these demonstrations have relied on relatively slow processes such as thermoelectric or joule heating, limiting data transfer rates to hundreds of bits per second (bps)^5^. While alternative approaches such as electrocaloric effect have the potential for faster modulation speeds of up to 100 MHz/K^6^, to date they have not exceeded hundreds of bps either^7^. Other methods of producing extremely fast thermal radiation, up to 10 GHz^8^, are limited to “bright” emission only and thus cannot be used in covert communications. Here we demonstrate a different approach to covert data communications^9^ which we call thermoradiative signatureless communication using the luminescence properties of mid-infrared (MIR) photodiodes, conceptually depicted in Fig. 1a. By modulating the bias on an MIR photodiode between forward (V > 0) and reverse (V < 0) to represent binary data, its net emission above the thermal background (V = 0) alternates between electroluminesence and a phenomena known as negative luminescence. By balancing these two states, the net emission becomes indistinguishable from the thermal backgoround by detectors with lower bandwidth. Negative luminescence under reverse bias^10–13^ is the symmetric process to forward biased electro-luminescence wherein the MIR semiconductor diode emits less radiation under reverse bias than it would at equilibrium. Interest in negative luminescence has been revived by a recent application of mid-infrared photodiodes as energy harvesting devices, known as thermoradiative diodes^14–19^. Furthermore, the negative luminescence effect is improved with photovoltaic pn-junction style photodiodes, and may not be not compatible with recent advances in photoconductive elements, although extremely fast modulation rates have been seen in advanced photoconductive device architectures^20^.

In this work, by balancing negative and electro-luminescence in thermoradiative MIR photodiodes, we demonstrate thermoradiative signatureless data rates up to 100 kbps and 1 MHz modulation frequencies while maintaining a time-averaged null signal compared to the thermal background, as confirmed using a low-bandwidth detector. The use of MIR photodiode emission over conventional thermal blackbody emission has several advantages: the lattice temperature of the device is not changed, which allows increased modulation speed and thus increased data transfer rates; narrowband emission enables multiplexing via encoding different information on different wavelength data channels; and better directionality of emission is possible e.g. through structured metasurfaces. We discuss further applications and potential advances in this technological space, including the use of meta-optics and 2D materials.

## Results

Here we present a relatively simple but illustrative demonstration of thermoradiative signatureless communications using the setup depicted in Fig. 1b. The two devices under study are mid-infrared (MIR) emitting HgCdTe (MCT) thermoradiative diodes at nominally 6 µm (PVI-6) or 10.6 µm (PVI-10.6). More details on the photodiodes and the experiment can be found in the “Materials and Methods”, and “Supplementary Information”.

**Fig. 2:**
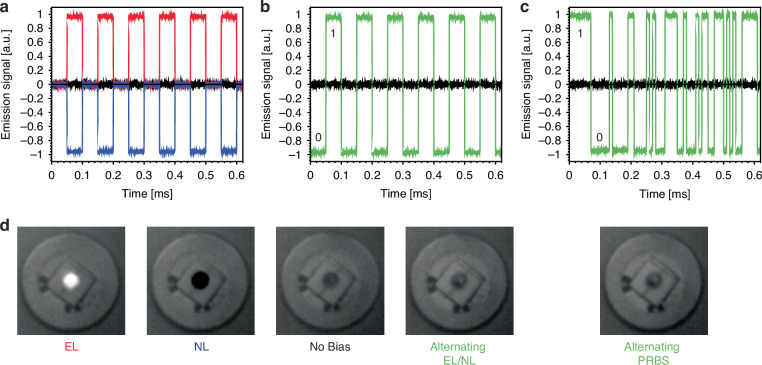
Experimental demonstration of thermoradiative signatureless communication. Detected emission signals from the PVI-6 photodiode when biased at (**a**) 50% duty cycle at 10 kHz and forward biased at 28 mV (red) compared to reverse biased at -81 mV (blue), (**b**) 50% duty cycle at 10 kHz with alternating bias (28 mV vs -81 mV) and no time-averaged thermal signature, and **c** pseudo random bit sequence (PRBS) at 100 kbps and no time-averaged thermal signature. Background subtracted response is in black. **d** Associated thermal camera images taken with a FLIR X6540sc camera at an integration time of 3.4 ms (covering the 4.9–5.5 µm spectral range)

Figure 2a depicts these results for the PVI-6 device using the oscilloscope feature, initially at a 50% duty and 10 kHz. It is clear that the bias voltage can be chosen to balance electro- and negative luminescence, albeit requiring a larger reverse bias of -81 mV to achieve the same signal as a forward bias of 28 mV. In Fig. 2b, the signal is alternated between electro- and negative luminescence directly. In this method, standard binary ‘0 s’ and ‘1 s’ can be encoded on the negative and electro-luminescence respectively while maintaining a time-average signal equivalent to the background thermal radiation. While the results in Fig. 2a–c are not shown at the maximum available negative luminescent signal magnitude available for this unoptimized negative luminescent device and given the lack of any electronic bandwidth filtering or averaging on the presented signal, the achieved signal-to-noise ratio of 67 is promising for this technique. To further demonstrate the data communication potential of this scheme, Fig. 2c shows a 100 kbps pseudo-random bit sequence to represent real data transfer. Thermal camera images in Fig. 2d further illustrate the absence of an optical signature of communications. It is clear that the effective radiation temperature for the photodiode is greatly increased compared to ambient when under forward bias and similarly cooled under reverse bias, as the electro- and negative luminescent signature of this device resides in the mid-infrared range between 4 and 6.5 µm and peaking at 5.3 µm (see the Supplementary Information for the spectral emission signature of the PVI-6 photodiode). But under the alternating forward and reverse bias or the pseudorandom bit sequence the thermal image is almost undistinguishable from that of the background given the relatively slow speed of the infrared camera. It should be noted that at high bias there can be a slight red shift of the electro-luminescence compared to the negative luminescence spectrum due to the Moss–Burstein effect^13,14^.

**Fig. 3:**
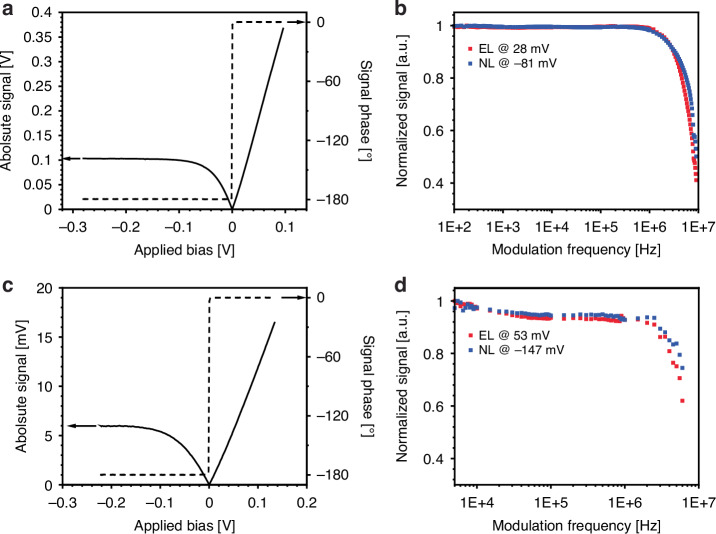
Bias and frequency dependent electroluminescence and negative luminescence. Detected emission signals from the PVI-6 (**a**, **b**) and PVI-10.6 (**c**, **d**) photodiodes as a function of voltage bias at 10 kHz and modulation frequency in both EL and NL modes at 50% duty cycle. For the PVI-10.6 photodiode measurement a 1 kHz longpass filter was used to reduce ambient noise

The bias and frequency dependent EL/NL signals can be seen for both the PVI-6 and PVI-10.6 photodiodes in Fig. 3. The emission signature of the PVI-10.6 photodiode peaks at 8.8 µm (see the Supplementary Information for the spectral emission signature of the PVI-10.6 photodiode). As expected, the emission signal follows a clear exponential-like diode dependence with bias as there is a limit to the suppression of thermal radiation in reverse bias. There is thus a limit on the magnitude of the signal that can be transmitted if EL and NL are kept equal as while electro-luminescence can in principle be continuously scaled upwards in forward bias while the device can handle the current, negative luminescence saturates in reverse bias. It is clear that the PVI-6 outperforms the PVI-10.6 in terms of flatness of its frequency response, although as expected from a lower bandgap device the NL signal from the PVI-10.6 requires a larger bias to saturate. However, the lower bandgap also induces significantly larger noise. Regardless, the -3 dB roll-off for the experiments occurred at 7 MHz for the PVI-6 and 5 MHz for the PIV-10.6, far in excess of previous thermal data transfer demonstrations^3–5^ while maintaining the capacity for signatureless communication. This frequency limit was imposed by a combination of the detectors (23 MHz and 11 MHz cut-off frequencies for the PVI-6 and PVI-10.6 respectively) and the 10 MHz clock of the function generator. Moreover, since the currents are much larger in EL operation as opposed to NL operation, the frequency roll off is likely governed primarily by the contacting of the devices themselves. Given recent advances in mid-infrared semiconductor emitters and photodetectors, a specifically designed emitter/reciever system could likely achieve significantly higher data transmission rates into the GHz^21^.

## Discussion

**Fig. 4:**
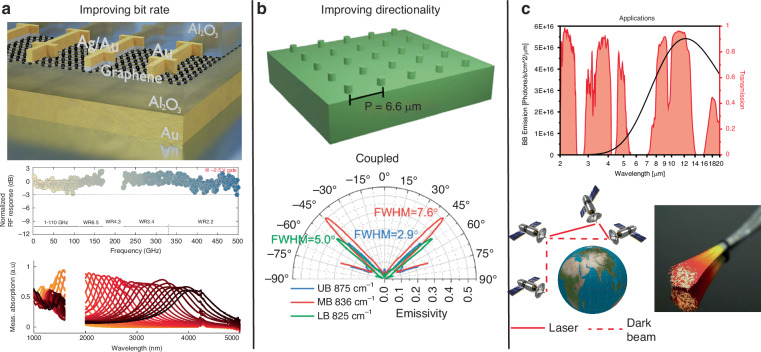
Future directions of thermoradiative signatureless communication. **a** Increasing bit rate. Here a metameterial graphene photodetector is shown to operate up to 500 GHz across part of the MIR spectrum (reproduced with permission from^26^, copyright 2023 AAAS). **b** Improving directionality such as through the use of metasurfaces. Here a 4H-SiC metasurface shows strong directionality of thermal emission (reproduced with permission from^34^, copyright 2021 American Chemical Society). **c** Applications. Terrestrial free space communications will be limited by the atmospheric transmission window (adapted with permission from^18^), but outer space inter-satillete communication will be limited primarily by directionality. MIR optical fibers enable long distance terrestrial covert communications without the issue of line of sight or atmospheric absorption

One of the most important advances necessary for thermoradiative signatureless communications to become practical is improving the data transfer speeds for both the emitter and the detector, as laid out in Fig. 4a. Commercial mid-infrared photovoltaic photodetector packages are already capable of up to GHz detection rates (HgCdTe, VIGO Systems), several orders of magnitude faster than what was demonstrated here. But for higher modulation speeds, one must look to develop alternative semiconductor photodiode materials. One option is the family of 2D materials^22^. Black phosphorus (bP), a promising 2D mid-infrared semiconductor, has already shown response bandwidths of 3 GHz at room temperature while retaining a responsivities comparable to conventional silicon photodiodes^23,24^. Graphene offers prospects for acheiving THz detection bandwidths. Due to small electron-phonon coupling in graphene, hot electron effects become dominant at the nanoscale, leading to efficient phototermoelectric generation^25^. This has already been exploited to realise ultrafast and high responsivity graphene photodetectors, with bandwidths of 500 GHz for photodetection in the near infrared^26^, and 95 GHz for THZ photodetection^27^. We propose that in mesoscopic graphene thermoelectric devices an electrical current could be used to directly modulate the electron temperature out of equilibrium with the lattice via the hot-electron Peltier effect, and thus modulate blackbody emission. Unlike prior demonstrations of thermal communications^3,5^ wherein the Peltier effect was used to modulate a body’s lattice temperature and emissivity, the ultimate speed of hotelectron Peltier devices is limited by the thermal relaxation time for electrons, which is on order 10 ps in graphene^27^. While the intrinsic absorption of graphene is only approximately 2.3% (regardless of wavelength), combining graphene with plasmonic resonances can enhance the absorptivity/emissivity to close to 100%^28^ and thus boost photon detection/emission.

Another requirement for free-space communcations will be to enhance the directionality of emission as outline in Fig. 4b. Conventional photodetectors commonly use an index matched hyperhemispherical immersion lens to both restrict the view factor (and thus emission cone), to 35 degrees in our case, and increase the effective optical area of the photodiode^29^. But this ultimately restricts the size of the photodiode to much smaller than the immersion lens, and thus the scalability of any emitter. An alternative to immersion lenses has been the development of planar nanophotonic metasurfaces^30^ or meta-lens arrays which can also be extended into the MIR^31–33^. The use of such resonant structures, especially in near-field proximity to the emitter, could result in a number of advantages. First, it has been shown that coupling to such a meta-surface can create a highly directional beam of only a few degrees divergence from an otherwise incoherent thermal source^34,35^. This comes with the added advantage of enabling narrowband emission^36^. Several different narrowband emitter/detectors would enable both wavelength-division multiplexing (WDM) schemes for further increasing data throughput as well as potentially providing further communications security. While the development by any one party of sources and detectors with the highest bandwidth would enable signatureless secure communications by that party, with only a single communications channel there is the concern that an observer with a sufficiently high bandwidth detector could view the communications. But specific protocols for multiplexing could be developed that also protect the security of communications. Finally, the presence of resonant structures has been used to enhance/control both the absorption from MIR photodetectors^37,38^ and emission from MIR sources^39–41^.

Using mid-infrared radiation for terrestrial free-space communications requires consideration of the atmospheric transmission windows, primarily at 3-5 µm and 8–14 µm, which limits the available wavelengths and distances for transmission^42^. Alternatively, as shown in Fig. 4c, space based communications relax these restrictions, but the potentially greater distances involved put even greater emphasis on improved directionality. In the short term, direct coupling of the emitter to a mid-infrared optical fiber, likely either chalcogenide glass^43^ or hollow core^44^, opens up the possibly of combining conventional telecommunications with thermoradiative signatureless communications as well as enabling long distance terrestrial covert communications without the issue of line of sight or atmospheric absorption. Fluoride optical fibers^45^ are especially attractive for relatively low transmission losses across both the telecoms and mid-infrared wavelength ranges. Combining covert and conventional telecommunications on the same transmission line also helps keep the use of the optical fiber for covert communications from being immediately obvious to an observer.

We have proposed and demonstrated a new form of covert and secure data transmission called thermoradiative signatureless communication via the balancing of negative luminescence and electro-luminescence in mid-infrared emitting HgCdTe thermoradative diodes. The balancing of reverse biased negative luminescence and forward biased electro-luminescence appears indistinguishable from the unbiased background emission of the devices to any detector lacking sufficient bandwidth to detect the modulation. With our existing setup we have demonstrated up to 100 kbps data rates and several MHz modulation frequencies near both the 3-5 µm and 8–14 µm atmospheric windows. While atmospheric absorption may limit free-space terrestrial communications, both outer space communication between satellites and land fiber based communications overcome this issue. Future prospects for this new form of covert communications will require improvements to both the bit rate and directionality. 2D materials such as graphene have the prospect of pushing the bandwidths for emission and detection to the THz regime, while metasurfaces promise both improved directionality as well as narrowband emission.

## Materials and methods

### Experimental details

The emission from a mid-infrared (MIR) emitting HgCdTe (MCT) thermoradiative diode (either PVI-6 or PVI-10.6 from VIGO System) held at 294 K (room temperature) was imaged with either a CaF_2_ or a ZnSe lens onto a 200 K cooled photodetector formed of nominally the same photodiode mounted on a VIGO PIP transimpedance pre-amplifier. Bias was applied to the emitting photodiode via an Agilent 33500B function generator and the resulting voltage time series from the photodetector after thermal background subtraction monitored with a Zurich HF2LI data acquisition unit.

## Supplementary information

Supplementary Information for “Balancing positive and negative luminescence for thermoradiative signatureless communications”

## Data Availability

Data is available from the authors upon reasonable request.
