# Supplementary material for: Balancing positive and negative luminescence for thermoradiative signatureless communications
Source: Light Sci Appl. 2026 Mar 5;15:148. doi: 10.1038/s41377-025-02119-y (PMC12963501; doi:10.1038/s41377-025-02119-y)
Supplement: Supplementary file 1 — Supplementary Information for “Balancing positive and negative luminescence for thermoradiative signatureless communications” [file 41377_2025_2119_MOESM1_ESM.pdf]

Supplementary Information for "Balancing positive and negative luminescence for thermoradiative signatureless communications"

Michael P. Nielsen[1*], Stefan A. Maier[2,3*], Michael S. Fuhrer[2*] and Nicholas J. Ekins-Daukes[1*]

[1]School of Photovoltaic and Renewable Engineering, UNSW Sydney, Kensington, 2052, NSW, Australia.

[2]School of Physics and Astronomy, Monash University, Clayton, 3800, Victoria, Australia.

[3]Department of Physics, Imperial College London, London, SW7 2AZ, U.K.

*Corresponding authors. E-mails: michael.nielsen@unsw.edu.au; stefan.maier@monash.edu; michael.fuhrer@monash.edu; nekins@unsw.edu.au;

Keywords: mid-infrared communication, thermoradiative diodes, negative luminescence

In the main text we report on two mid-infared HgCdTe photodiodes from VIGO Systems: PVI-6-1x1-TO39-wNone-36 and PVI-10.6-0.5x0.5-TO39-wNone-36. In both cases they are packaged in a TO39 can and have a integrated hyperhemispherical GaAs immersion lens that limits their field of view to 36 degrees. Figure S1 depicts the steady state device results for both devices including the room temperature external quantum efficiency (EQE) and dark IV (and thus dynamic resistance close to zero bias). In addition, spectral regions of interest are also shown. For the PVI-6 device, Figure S1c reproduces the measured electroluminescence and negative luminescence from [1]. Highlighted in grey is the spectral region the relevant thermal camera for Figure 2 was sensitive to.

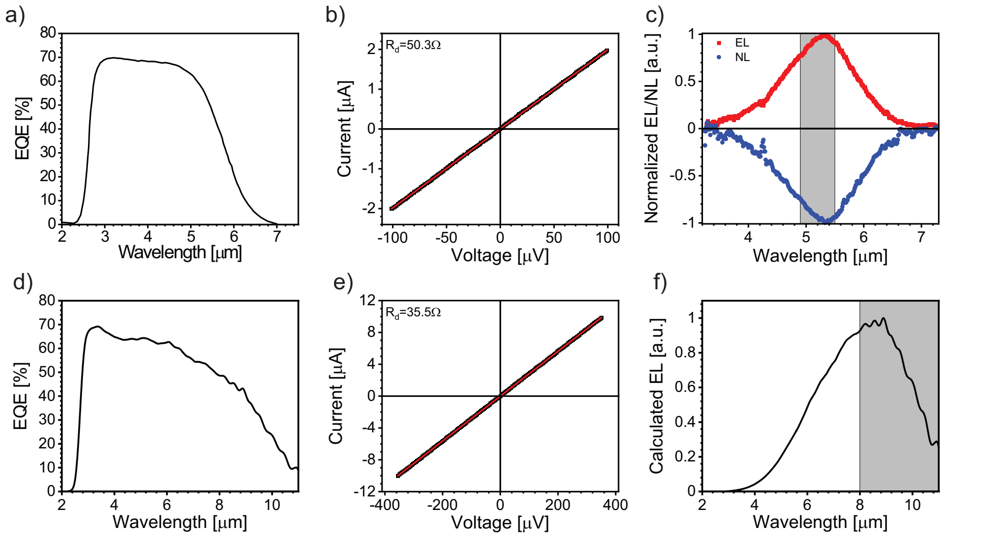

Fig. S1 Device characteristics including external quantum efficiency (EQE) and dark IV for the PVI-6 (a,b) and PVI-10.6 (d,e) diodes. (c) Measured normalized electroluminescence and negative luminescence spectra for the PVI-6 photodiode reproduced from [1], with the greyed area showing the coverage of the FLIR X6540sc camera. (f) Normalized electroluminescence spectrum for the PVI-10.6 photodiode calculated from the normal incidence EQE using the generalized Planck law, with the greyed area showing the coverage of the InfiRay P2 Pro camera.

For the PVI-10.6 device, in Figure S1f we instead calculate the electroluminescence spectra from the normal incidence EQE using the generalized Planck law, with again the relevant thermal camera region highlighted in grey.

Figure S2 depicts the demonstration of thermoradiative signatureless communciations for the PVI-10.6 device using the oscilloscope feature, initially at a 50% duty and 10 kHz, following that presented for the PVI-6 device in Figure 2 of the main text. It is clear that the bias voltage can be chosen to balance electro- and negative luminescence, albeit requiring a large reverse bias of -147 mV to achieve the same signal as a forward bias of 53 mV. Compared to the PVI-6 device, and consistent with the results of Figure 3 of the main text, the background noise associated with using this

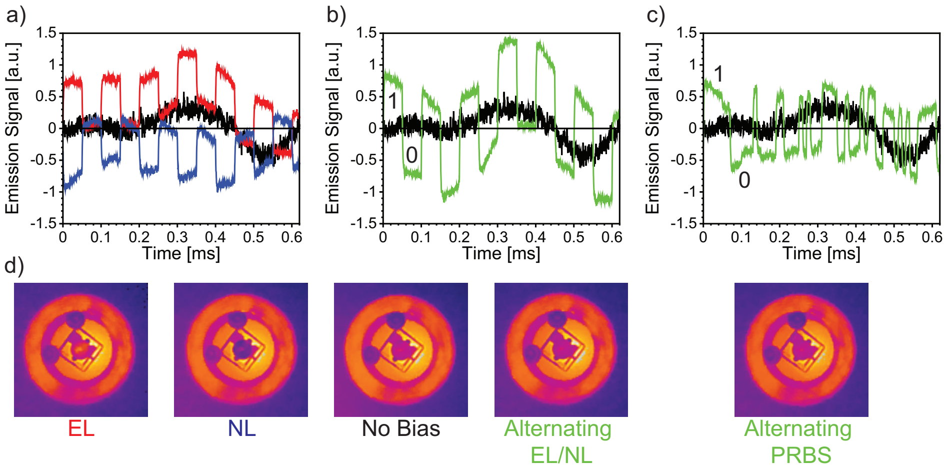

Fig. S2 Detected emission signals from the PVI-10.6 photodiode when biased at (a) 50% duty cycle at 10kHz and forward biased at 53 mV (red) compared to reverse biased at -147 mV (blue), (b) 50% duty cycle at 10 kHz with alternating bias (53 mV vs -147 mV) and no time-averaged thermal signature, and (c) pseudo random bit sequence at 100 kbps and no time-averaged thermal signature. Background response is in black. (d) Associated thermal camera images taken with a InfiRay P2 Pro camera at 25 Hz refresh rate (covering the 8-14 µm spectral range).

much lower bandgap semiconductor is much worse. But it remains that the modulated signal, both at 10 KHz with a 50% duty cycle and a pseudo random bit sequence at 100 kbps, show no time-averaged thermal signature as evidenced by thermal camera images.

References

[1] Nielsen, M.P., Pusch, A., Sazzad, M.H., Pearce, P.M., Reece, P.J., Ekins-Daukes, N.J.: Thermoradiative power conversion from HgCdTe photodiodes and their current–voltage characteristics. ACS Photonics **9**(5), 1535–1540 (2022) https://doi.org/10.1021/acsphotonics.2c00223
